# Fugitive medical and patient-derived aerosol particle distribution following heparin nebulization in patients with COVID-19 acute hypoxemic respiratory failure: a secondary analysis of the CHARTER study

**DOI:** 10.1186/s40635-024-00659-y

**Published:** 2024-08-26

**Authors:** Michael Walsh, Marc Mac Giolla Eain, Ronan MacLoughlin, John Laffey, Bairbre McNicholas

**Affiliations:** 1 Department of Anaesthesia and Intensive Care Medicine, University Hospital Galway, Newcastle Road, Galway, H91 YR71 Ireland; 2 Research and Development, Science and Emerging Technology, Aerogen Ltd., Galway Business Park, Galway, Ireland; 3 Anaesthesia and Intensive Care Medicine, School of Medicine, University of Galway, Galway, Ireland

**To the Editor,**

Aerosolisation is an effective delivery mechanism for direct delivery of therapeutics into the lung and has the potential to reduce systemic side effects. However, the potential for and extent of environmental contamination associated with aerosolisation needs to be determined, particularly whether this is influenced by oxygen delivery devices. The aim of this study was to determine the distribution of fugitive medical and patient-derived aerosols around the bed spaces of patients undergoing the management of COVID-19 pneumonia and determine differences in the aerosol particle distribution based on device used for respiratory support. A convenience sample of patients from the ‘Can Nebulised HepArin Reduce acuTE lung injury in Patients with SARS-CoV-2 Requiring Mechanical Ventilation in Ireland (CHARTER-Ireland)’ study, a randomized-controlled trial of nebulized unfractionated heparin in ICU patients with SARS-CoV-2 requiring advanced respiratory support, participated in this research [1]. An optical particle sizer (OPS 3300, TSI, Inc., USA) with an inflow hose was attached to the sampling port at the nursing station near the patients' bed space. This setup measured fugitive aerosols (FA) generated over a 24-h period during which the patient was enrolled. Measurements were recorded at 1-min intervals throughout the 24 h. Details on background, ethics, and methodology are outlined in the supplementary appendix. A total of 20 separate periods of air sampling collection were recorded on 12 patients, of which 14 were episodes from patients randomized to the heparin treatment and 6 to standard care. All patients underwent advanced respiratory support with 10 managed with high-flow nasal cannula oxygen (HFNO), 7 managed with continuous positive airway pressure (CPAP), and 3 managed with invasive mechanical ventilation (IMV). Details on patients and outcomes are outlined in supplementary Table 1. Peak particle mass concentrations were significantly higher for patients on HFNO compared to CPAP and IMV [23(4.3–37), 1.7 (0.2–4), 0.98 (0.7–2.9) µg/m^3^, respectively, *p* < 0.0001] (Fig. 1A). The median particle mass concentration over a 24-h period was also higher in the HFNO group compared to CPAP and IMV [24.6(17.2–35.5), 2.1 (0.1–28.3) vs. 3.3 (1.6–7.8) µg/m^3^, *p* = 0.0008)], with no statistically significant differences between CPAP and IMV (*p* = 0.9). In the group of patients receiving nebulised heparin, there was no difference in median peak particle concentration over a 24-h period in those administered heparin compared to standard care (36.1(8.4–56.3) vs. 12.6(4.7–25.5) µg/m^3^, *p* = 0.1). In all patient groups receiving nebulized heparin, there was no difference in peak particle concentration during periods of heparin nebulisation, compared to periods between treatments (23.1 (4.3–36.8) vs. 21.8(14–25.2) µg/m^3^, *p* = 0.7) nor was there a different in median peak particle concentration over a 24 h period in those receiving aerosolized heparin vs standard care for HFNO, NIV, and IMV, respectively (Fig. 1B). In conclusion, there were significant differences in FA generation depending on the type of respiratory support, showing that use of HFNO therapy results in higher FA generation compared to CPAP and IMV. Reassuringly, the nebulization of heparin did not increase FA levels compared to standard care.

**Fig. 1:**
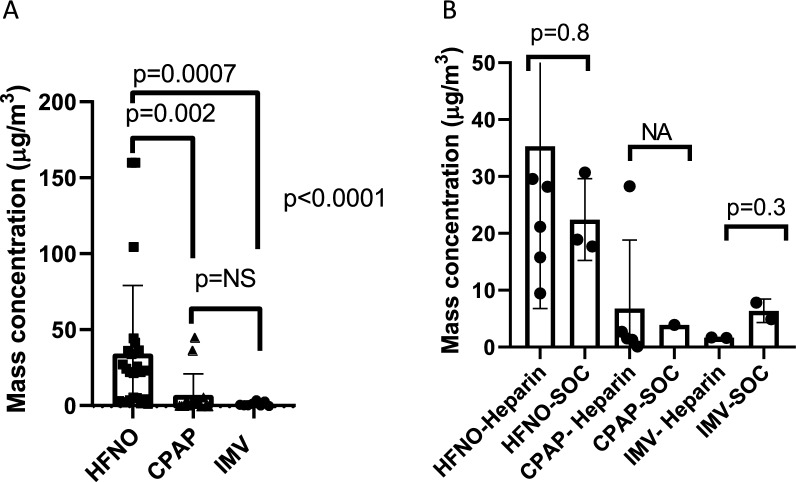
**A** Peak fugitive aerosol mass concentrations detected during heparin nebulisation for patients receiving nebulised heparin with high-flow nasal oxygen (HFNO), continuous positive airway pressure (CPAP), and invasive mechanical ventilation (IMV). *NS *non-significant. Kruskal–Wallis test followed by Dunn's multiple comparisons correction. **B** Average particle mass concentration over a 24-h period for patients receiving nebulised heparin compared to standard of care (SOC) with high-flow nasal oxygen (HFNO),continuous positive airway pressure (CPAP), and invasive mechanical ventilation (IMV). Kruskal–Wallis test followed by Dunn's multiple comparisons correction

### Supplementary Information

Supplementary Material 1.

## Data Availability

Data are available to investigators on reasonable request; please email lead author.

## Abbreviations

SOC: Standard of care
ICU: Intensive Care Unit
HFNO: High flow nasal cannula oxygen
IMV: Invasive mechanical ventilation
CPAP: Continuous positive airway pressure
FA: Fugitive aerosols
OPS: Optical particle sizer
CHARTER: Can Nebulised HepArin Reduce acuTE lung injury in Patients with SARSCoV-2 Requiring Mechanical Ventilation in Ireland (CHARTER)
VMN: Vibrating mesh nebuliser
