# Supplementary material for: Fugitive medical and patient-derived aerosol particle distribution following heparin nebulization in patients with COVID-19 acute hypoxemic respiratory failure: a secondary analysis of the CHARTER study
Source: Intensive Care Med Exp. 2024 Aug 26;12:73. doi: 10.1186/s40635-024-00659-y (PMC11347535; doi:10.1186/s40635-024-00659-y)
Supplement: Supplementary file 1 — Supplementary Material 1. [file 40635_2024_659_MOESM1_ESM.docx]

**Background**

In the clinical setting, nebulisers aerosolise drugs to concentrate therapeutic agents within the lungs, thereby attenuating the potential for adverse systemic effects, and/or focusing the therapeutic within the lung compartment. In intensive care settings, patients requiring nebulised pharmacotherapy often require simultaneous advanced respiratory support. The evidence base for the efficacy of aerosolized medications is developing rapidly, with efficacy demonstrated for inhaled amikacin in preventing ventilator associated pneumonia a recent example [1]. In addition, aerosolized delivery of complex advanced medical therapeutics, such as cell and gene-based therapies [2-6].

Equipment employed for aerosol generation and delivery are susceptible to leaks into the ambient environment. Such particles may emanate from aerosol not deposited on respiratory epithelium and then subsequently exhaled, or from breaches within the medical equipment - be it the ventilator, nebuliser, or patient interface. These extraneous particles, known as 'fugitive aerosols,' (FA) are heterogeneous in composition; they may consist of the pharmacological agent being administered (classified as 'medical') or derive from biological fluids secreted by the pulmonary epithelial lining (classified as 'patient-derived'). The inadvertent dissemination of fugitive particles in clinical environments poses potential risks to healthcare professionals and other patients who may be incidentally exposed. The implications of such exposure are contingent upon the volume and composition of the fugitive particles. The spectrum of consequences spans from the transmission of infections, as underscored by the COVID-19 pandemic, to inadvertent drug exposure.

Clinically relevant data emanating from actual hospitalised patients remains scarce. While a limited body of studies, particularly those involving healthy volunteers, suggests no marked difference in aerosol generation with specific oxygen delivery systems, other studies suggest a divergent trend, especially when evaluating patients with extant respiratory pathologies. One study delineated a notably elevated risk of developing new-onset asthma among respiratory therapists compared with physiotherapists following the commencement of their professional duties [7]. Similarly, an increased risk of asthma was noted ^[[1]](#endnote-1)^n newly practicing nurses in contrast to administrative and clerical personnel [8]. This increased incidence of occupational asthma incidence has been posited to be linked to the exposure to FA’s [9]. Additionally, it has been raised that exposure to nebulised antibiotics could inadvertently foster antibiotic resistance among those exposed [10].

In this study, we hypothesised that high flow nasal oxygen (HFNO) therapy would generate higher levels of FA emission compared to non-invasive or invasive ventilation, and that nebulisation of medications would increase the burden of FA emissions. To test these hypotheses, we examined FAs generated by a cohort of patients enrolled in a randomized phase 1b/2a trial exploring the efficacy of nebulised heparin in the management of COVID-19 induced Acute Respiratory Distress Syndrome (ARDS).

**Methods**

**Ethics**

Ethical approval for this study was secured from the National Research Ethics Committee (20-NREC-COV-104) and the Health Products Regulatory Authority (HPRA) of Ireland under the reference CT0900/650/001 Heparin Sodium. Prior to the inclusion of participants in the study, informed consent was obtained. In instances where individuals were incapacitated due to the severity of their illness, assent was sought and obtained from the next of kin.

**Participants**

The patient cohort for this study comprised a sample size of twelve (n=12) patients, all of whom were participants in a multi-centre phase II, randomized, open-label controlled trial, enrolled at our institution. The trial was designed to evaluate the therapeutic impact of nebulized unfractionated heparin on ICU patients afflicted with SARS-CoV-2 and necessitating advanced respiratory support (see full study protocol here) [11]. The patients were stratified into two distinct cohorts: an intervention group, which received nebulized heparin at six-hour intervals in conjunction with the standard care regimen, and a control group, which was administered the conventional standard of respiratory care.

**Nebuliser setup**

In this study, we employed the Aerogen Solo™ (Aerogen Ltd., Ireland), a vibrating mesh nebulizer, for the administration of nebulized heparin. It was also utilized consistently if patients required nebulization of additional medications as part of their standard respiratory care (SOC). The nebuliser is placed on the inspiratory limb near the Y-connector, after the filter which is placed between the ventilator and the nebuliser. To prevent interaction between medications, nebulized heparin was administered separate from other medications, at a dosage of 25,000 Units in 5 mls every six hours. For intubated patients, the nebulizer was positioned in the inspiratory limb of the ventilator circuit. This configuration was chosen to ensure optimal delivery of medication to the patient while minimizing the potential for FA generation. All patients receiving CPAP had either helmet or mask removed and nebuliser administered.

**FA measurement**

The optical particle sizer (OPS 3300, TSI, Inc., USA) was used to measure FA particles ranging from 0.3 to 10 micrometres. The OPS was situated on a metal tray at the patient's room to capture a representative sample of the air quality in the immediate vicinity of the healthcare setting. An inflow hose was affixed to the sampling port of the OPS and extended towards the nurses' computer station in the room (figure 1) and was approximately 1.5 – 2 m in distance from patient’s bedside. Particle mass concentrations (µg/m^3^) were recorded at one-minute intervals over a full 24-hour cycle.

**Clinical data collection**

Clinical data pertaining to the patients was recorded with precise time stamps in the electronic health record (EHR) by the attending nurse throughout the 24-hour sampling interval. The data recorded included the type of advanced respiratory support administered, the duration of said treatment, and any significant interventions conducted during the sampling period, such as diagnostic imaging, phlebotomy, and alterations in patient positioning. After the sampling period, this data was extracted from the EHR for comprehensive analysis. Correlations between recorded clinical events and fluctuations in the OPS particle concentration graph were examined for potential causality for observed spikes in the particle count. Baseline demographic and clinical data, initially compiled for the CHARTER study, were assimilated into this research for contextual analysis.

**Statistical analysis**

The statistical analysis of particle mass concentrations was conducted using GraphPad Prism (Version 8, USA). The Kolmogorov-Smirnov test was utilized to determine the normality of the continuous data distribution. Data adhering to normal distribution parameters were subjected to the Student’s t-test for two groups or a one-way ANOVA test for greater than two groups, while non-parametric data were evaluated using the Mann-Whitney U test or Kruskal-Wallis test for greater than two groups. Dunn’s test was used for multiple comparisons. Mean and SD or median and interquartile range (IQR)I are reported where applicable. A p-value threshold of less than 0.05 was designated as the level for statistical significance.

**Discussion**

This study examined FAs from ICU patients' bed spaces, during treatment with nebulized heparin and advanced respiratory support for COVID-19 pneumonia. Nebulized drug therapy did not lead to an increase in FA detected using an optical particle analyser. In contrast, HFNC treatment led to higher levels of FAs compared to patients managed with CPAP or IMV. The lowest FA particle counts detected was in patients managed with IMV. The use of nebulized heparin as a therapy did not appear to increase the quantity of FAs detected in this study.

Our study demonstrates that the generation of FAs is dependent on the type of respiratory support therapy device used. The elevated particle counts observed in patients on HFNO may be attributable to multiple factors including higher oxygen flow rate, higher humidification of ambient and patient-emitted air. Of note, patients on continuous positive airway pressure ventilation (CPAP) were switched to high-flow nasal cannula (HFNC) only for drug administration and activities such as mouth care and diet, and were otherwise managed with a facemask or helmet. It is possible that FA detected during CPAP use were lower than that detected during HFNC treatment, as airflow was directed at the patients face and likely contained on the mask’s surface.

A study of 25 healthy volunteers found HFNO to generate more FAs than the baseline activities, however, the introduction of a filter substantially mitigated this effect. They also found that FA generated from HFNO were in the same size range, whether sampled directly from the HFNO device or from the exhaled breath of the user. Additionally, coughing produced the highest concentration of aerosols suggesting that the aerosol emission risks associated with HFNO and CPAP are comparable to those posed by natural respiratory events such as coughing or sneezing [12]. Consistent with this, Gaeckle *et al*. did not observe a significant rise in expired aerosol levels with the use of HFNO or CPAP in healthy individuals but an increase in FA levels was measured from patients suffering from viral pneumonia which they felt could be related to cough and mucus production [13].

In contrast to HFNC, mechanical ventilators are engineered to function as closed systems. FAs during IMV emanate from a limited number of sources: the ambient environment, the ventilator’s exhalation port, potential leaks in the circuit, or the patient's upper airway, specifically proximal to the endotracheal (ET) tube cuff. Contemporary ventilators are designed with minimal leak rates and include alarm systems to alert healthcare operators in the event of a leak. For the purposes of this experiment, the IMV configuration incorporated a vibrating mesh nebulizer (VMN) within the inspiratory limb of the ventilation circuit. Additionally, a filter was employed at the expiratory port, serving to capture any aerosols exhaled by the patients. This is supported by existing *in-vitro* research on FAs during IMV. For instance, one study utilized Schlieren imaging techniques alongside a simulated head profile to investigate the dynamics of air and aerosol particle movement during IMV under various aerosol configurations, noting an absence of FA release during nebulization when a closed-circuit VMN was used [14].

We found no increase in the quantity of FAs among patients treated with nebulized heparin in comparison to those who were treated with standard of care across the various respiratory devices utilised in this study. Several factors could explain the absence of increased FAs in the heparin group. Firstly, the utilization of the VMN for heparin delivery is known to emit the least amount of FAs among nebulizer variants [15-17] . For patients on IMV, the integration of a Servo Duo Guard with a high-efficiency particulate air filter in the expiratory limb might have effectively curtailed the escape of FAs during heparin nebulization. Available data on FAs pertain to medications such as albuterol among others, which possess different physicochemical properties compared to heparin and are known to affect aerosol behaviour. Heparin is a large polysaccharide with a molecular weight ranging from 3,000 to 30,000 Da. Particles of larger molecular size generally form larger aerosols, which might be less likely to remain airborne and tend to settle more rapidly. Additionally, the larger molecules may be more effectively absorbed or entrapped by respiratory mucus, reducing environmental leakage. Our sampling apparatus was positioned 1 - 1.5 meters from the patient, where the healthcare worker would usually be positioned; hence, any large FAs of unfractionated heparin that may have been emitted could have settled before reaching the sampling inlet.

Our study had several limitations. Firstly, we were unable to quantify the proportion of particles that were either bioaerosols or medical aerosols and size would not distinguish between medical and patient derived. This renders it challenging to assess the risk of bystander exposure, as the repercussions depend on the specific type of particle to which one is exposed. Moreover, strategies for future mitigation might vary according to the predominant particle type produced by one form of respiratory support. This limitation could be addressed in subsequent studies through the use of the built-in filter in the OPS with lab analysis of collected particles, or by examining particle size distributions built-in to the OPS device. Secondly, we did not investigate how the use of various CPAP patient interfaces or variations in HFNO settings, such as flow rate, which affects the quantity and deposition of aerosol particles in the lungs, might influence the dispersion of FA particles. Thirdly, our sample size was relatively small; some patients underwent multiple test periods, and the number of tests was not uniformly distributed across different forms of respiratory support, potentially skewing the results. Fourthly, while a strength of this study is that it was conducted in a clinical setting, this is inherently less controlled than a laboratory setting. This meant certain variables that could influence aerosol concentrations could not be controlled for and likely led to large variance, standard deviations, in the peak and average data. In the clinical setting, there is movement of staff, patients, and bystanders in and around both the patient and nurses’ station, where the OPS was located, during the normal working day which could affect the measurements being taken and should be taken into consideration when analysing the data.

Finally, we only measured optical density and lacked data on the clinical implications of the generated aerosols, such as infective transmissibility or healthcare worker exposure to heparin. These are critical factors for clinical interest. Future studies should determine if the drug levels detected on surfaces through swabbing correlate with optical particle density or if captured particles can be further analyzed using mass spectrometry to identify their constituents.

In conclusion, the study did detect significant differences in FA generation depending on the type of respiratory support, showing that use of HFNO therapy results in higher FA generation compared to CPAP and IMV and nebulization of heparin does not result in an increase in FA levels compared to standard care. To our knowledge, no other research has specifically investigated FA emissions resulting from drug nebulization and we would expect this to be generalizable to other drugs. The implications of this research will contribute to the development of policies and the refinement of best practices aimed at mitigating the risks inherent to inadvertent aerosol releases within healthcare settings.

**References**

1. Ehrmann S, Barbier F, Demiselle J, Quenot JP, Herbrecht JE, Roux D, Lacherade JC, Landais M, Seguin P, Schnell D *et al*: **Inhaled Amikacin to Prevent Ventilator-Associated Pneumonia**. *N Engl J Med* 2023, **389**(22):2052-2062.

2. McCarthy SD, Horgan E, Ali A, Masterson C, Laffey JG, MacLoughlin R, O'Toole D: **Nebulized Mesenchymal Stem Cell Derived Conditioned Medium Retains Antibacterial Properties Against Clinical Pathogen Isolates**. *J Aerosol Med Pulm Drug Deliv* 2020, **33**(3):140-152.

3. Gonzalez HE, McCarthy SD, Masterson C, Laffey JG, MacLoughlin R, O'Toole D: **Nebulized mesenchymal stem cell derived conditioned medium ameliorates Escherichia coli induced pneumonia in a rat model**. *Front Med (Lausanne)* 2023, **10**:1162615.

4. McCarthy SD, Tilbury MA, Masterson CH, MacLoughlin R, Gonzalez HE, Laffey JG, Wall JG, O'Toole D: **Aerosol Delivery of a Novel Recombinant Modified Superoxide Dismutase Protein Reduces Oxidant Injury and Attenuates Escherichia coli Induced Lung Injury in Rats**. *J Aerosol Med Pulm Drug Deliv* 2023, **36**(5):246-256.

5. MacLoughlin RJ, Higgins BD, Devaney J, O'Toole D, Laffey JG, O'Brien T: **Aerosol-mediated delivery of AAV2/6-IkappaBalpha attenuates lipopolysaccharide-induced acute lung injury in rats**. *Hum Gene Ther* 2015, **26**(1):36-46.

6. Green O, Shenberg G, Baruch R, Argaman L, Levin T, Michelson I, Hadary R, Isakovich B, Golos M, Schwartz R *et al*: **Inhaled Exosomes Genetically Manipulated to Overexpress CD24 (EXO-CD24) as a Compassionate Use in Severe ARDS Patients**. *Biomedicines* 2023, **11**(9).

7. Kern DG, Frumkin H: **Asthma in respiratory therapists**. *Ann Intern Med* 1989, **110**(10):767-773.

8. Arif AA, Delclos GL, Serra C: **Occupational exposures and asthma among nursing professionals**. *Occup Environ Med* 2009, **66**(4):274-278.

9. Dimich-Ward H, Wymer ML, Chan-Yeung M: **Respiratory health survey of respiratory therapists**. *Chest* 2004, **126**(4):1048-1053.

10. Daniels LM, Juliano J, Marx A, Weber DJ: **Inhaled Antibiotics for Hospital-Acquired and Ventilator-Associated Pneumonia**. *Clin Infect Dis* 2017, **64**(3):386-387.

11. Sheehan JR, Calpin P, Kernan M, Kelly C, Casey S, Murphy D, Alvarez-Iglesias A, Giacomini C, Cody C, Curley G *et al*: **The CHARTER-Ireland trial: can nebulised heparin reduce acute lung injury in patients with SARS-CoV-2 requiring advanced respiratory support in Ireland: a study protocol and statistical analysis plan for a randomised control trial**. *Trials* 2022, **23**(1):774.

12. Hamilton FW, Gregson FKA, Arnold DT, Sheikh S, Ward K, Brown J, Moran E, White C, Morley AJ, Group A *et al*: **Aerosol emission from the respiratory tract: an analysis of aerosol generation from oxygen delivery systems**. *Thorax* 2022, **77**(3):276-282.

13. Gaeckle NT, Lee J, Park Y, Kreykes G, Evans MD, Hogan CJ, Jr.: **Aerosol Generation from the Respiratory Tract with Various Modes of Oxygen Delivery**. *Am J Respir Crit Care Med* 2020, **202**(8):1115-1124.

14. Mac Giolla Eain M, Joyce M, MacLoughlin R: **An in vitro visual study of fugitive aerosols released during aerosol therapy to an invasively ventilated simulated patient**. *Drug Deliv* 2021, **28**(1):1496-1500.

15. O'Toole C, McGrath JA, Joyce M, O'Sullivan A, Thomas C, Murphy S, MacLoughlin R, Byrne MA: **Effect of nebuliser and patient interface type on fugitive medical aerosol emissions in adult and paediatric patients**. *Eur J Pharm Sci* 2023, **187**:106474.

16. Joyce M, McGrath JA, Mac Giolla Eain M, O'Sullivan A, Byrne M, MacLoughlin R: **Nebuliser Type Influences Both Patient-Derived Bioaerosol Emissions and Ventilation Parameters during Mechanical Ventilation**. *Pharmaceutics* 2021, **13**(2).

17. O'Toole C, Joyce M, McGrath JA, O'Sullivan A, Byrne MA, MacLoughlin R: **Fugitive aerosols in the intensive care unit: a narrative review**. *Ann Transl Med* 2021, **9**(7):592.

1. **Supplementary Table 1**. Descriptive data of patient characteristics.

   |  | **Overall** | **IMV** | **HFNO** | **CPAP** |
   | --- | --- | --- | --- | --- |
   | ***n* Patients** | 12 | 3 | 5 | 4 |
   | **Age (years, range)** | 54 [48, 66] | 69 [49, 81] | 53 [43, 54] | 62 [48,70] |
   | **Sex**  **Male**  **Female** | 7 (58)  5 (42) | 1 (33)  2 (66) | 3 (60)  2 (40) | 3 (75)  1 (25) |
   | **Number of 24hr OPS Tests** | 20 | 3 (15) | 10 (50) | 7 (35) |
   | **Number of tests per patient** | 2 [1, 2] | 1 [1] | 2 [2, 3] | 2 [1, 2] |
   | **Number with/without heparin nebulisation** | 14/6 | 2/2 | 7/3 | 5/1 |
   | **Day of ICU admission when test performed** | 2 [2, 4] | 5 (2, 7) | 2 (1, 5) | 2 (1, 7) |
   | **Tests during nebulised heparin** | 16 (80) | 2 (66) | 7 (70) | 7 (72) |
   | **Outcomes 60 Day**  **Remain in ICU**  **Discharged ICU remain in hospital**  **Discharged from Hospital**  **Deceased** | 0  1 (8)  9 (75)  2 (17) | 0  0  2 (17)  1 (8) | 0  0  5 (42)  0 | 0  1 (8)  2 (17)  1 (8) |

   Table 1. Data are shown as median [Q25, Q75] or as n-number (%). Values are rounded to nearest whole number. OPS optical particle sizer. [↑](#endnote-ref-1)
